# A deep learning method to predict ankle joint moment during walking at different speeds with ultrasound imaging: A framework for assistive devices control

**DOI:** 10.1017/wtc.2022.18

**Published:** 2022-09-06

**Authors:** Qiang Zhang, Natalie Fragnito, Xuefeng Bao, Nitin Sharma

**Affiliations:** 1 Joint Department of Biomedical Engineering, North Carolina State University, Raleigh, NC, USA; 2 Joint Department of Biomedical Engineering, The University of North Carolina at Chapel Hill, Chapel Hill, NC, USA; 3 Biomedical Engineering Department, University of Wisconsin-Milwaukee, Milwaukee, WI, USA

**Keywords:** convolutional neural network, deep learning, human intent, human walking, plantarflexion, ultrasound imaging

## Abstract

Robotic assistive or rehabilitative devices are promising aids for people with neurological disorders as they help regain normative functions for both upper and lower limbs. However, it remains challenging to accurately estimate human intent or residual efforts non-invasively when using these robotic devices. In this article, we propose a deep learning approach that uses a brightness mode, that is, B-mode, of ultrasound (US) imaging from skeletal muscles to predict the ankle joint net plantarflexion moment while walking. The designed structure of customized deep convolutional neural networks (CNNs) guarantees the convergence and robustness of the deep learning approach. We investigated the influence of the US imaging’s region of interest (ROI) on the net plantarflexion moment prediction performance. We also compared the CNN-based moment prediction performance utilizing B-mode US and sEMG spectrum imaging with the same ROI size. Experimental results from eight young participants walking on a treadmill at multiple speeds verified an improved accuracy by using the proposed US imaging + deep learning approach for net joint moment prediction. With the same CNN structure, compared to the prediction performance by using sEMG spectrum imaging, US imaging significantly reduced the normalized prediction root mean square error by 37.55% (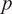 < .001) and increased the prediction coefficient of determination by 20.13% (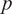 < .001). The findings show that the US imaging + deep learning approach personalizes the assessment of human joint voluntary effort, which can be incorporated with assistive or rehabilitative devices to improve clinical performance based on the assist-as-needed control strategy.

## 1. Introduction

The human ankle plantarflexor muscles play an essential role in the lower limbs’ activities of daily living. For example, they provide primary mechanical propulsion force in both forward and upward directions during walking, which is referred to as the “push-off” during the late stance phase of the gait cycle (Huang et al., 2015b; Zhang et al., 2022b). Weakened function or dysfunction of human ankle plantarflexor muscles due to neurological disorders and injuries such as stroke, multiple sclerosis, and spinal cord injury, can cause significant impairment of normal walking function, like decreased plantarflexion propulsion force, inefficient ankle mechanical power production, and reduced walking efficiency (Nuckols et al., 2020). Traditional treatment for ankle plantarflexion weakness primarily depends on physical therapy, which is labor-intensive and limited by the availability of clinical professionals (Kirshblum et al., 2007; Langhorne et al., 2011; Deng et al., 2018).

Current technological advancements to combat loss of lower limb joint function include neurorehabilitative treatments such as powered exoskeletons (Ferris et al., 2005; Jamwal et al., 2012; Huo et al., 2014; Murray et al., 2014; Jackson and Collins, 2015; Meng et al., 2016; Young and Ferris, 2016; Esquenazi et al., 2017; Zhang et al., 2017), functional electrical stimulation (Bajd et al., 1999; Mushahwar et al., 2007; Sharma et al., 2009; Kirsch et al., 2017; Alibeji et al., 2018b), soft exosuits (Bae et al., 2015; Awad et al., 2017; Siviy et al., 2020), and hybrid neuroprosthesis (Ha et al., 2016; Alibeji et al., 2018a; Kirsch et al., 2018; Tamburella et al., 2020; Molazadeh et al., 2021). Although these advancements have gained promising progress in helping people regain or enhance the lower limb’s joint functionalities, most of these approaches did not consider the wearers’ volitional effort or motion intent. However, a reliable estimation of volitional effort or motion intent is essential for more intuitive and transparent control method design, like assist-as-need (AAN) control (Pehlivan et al., 2016; Shahbazi et al., 2016). The AAN control-based rehabilitation methods often require the continuous estimation of human muscle force, joint moment, or human-machine interaction through measuring or combining modalities such as neural signals of the central nervous system (CNS), muscle contraction activities, joint kinematics, and joint kinetics (Lobo-Prat et al., 2014; Zhang et al., 2020a).

Existing continuous motion intent or volitional effort prediction approaches can roughly be divided into two categories: mechanical-type and biological-type. Mechanical-type approaches usually directly measure the physical interaction between the human and machine through force or torque sensors (Veneman et al., 2007; Huang et al., 2015a; Losey et al., 2018), or measure the motion intent based on IMU sensors (Baghdadi et al., 2018; Su et al., 2019). However, the susceptibility to undesired interaction is inevitable because of the misalignment between the human joint rotation center and the sensor’s fixture rotation center (Zanotto et al., 2015; Gopura et al., 2016). In addition, mechanical-type approaches cannot reveal the physiological changes *in* *vivo*. In contrast, biological-type non-invasive approaches, like surface electromyography (sEMG) and ultrasound (US) signals, are capable of assessing the generated neuromuscular force or joint torque. Given the fact that 30–150 ms prior to the corresponding motion is generated, neuromuscular signals can reflect human intent or volitional effort without any information delay or loss (Huo et al., 2014; Ding et al., 2016; Zhang et al., 2021). The continuous motion intent or volitional effort prediction by using neuromuscular signals usually depends on nonlinear dynamic models, like Hill-type neuromuscular model (HNM) (Lloyd and Besier, 2003; Besier et al., 2009; Sartori et al., 2012; Sartori et al., 2014; Ao et al., 2017; Dick et al., 2017; Zhang et al., 2020a, b), that builds the correlated mapping between the input neuromuscular signals and the output muscle force, joint moment, or joint motion. However, the nonlinear model consists of many physiological parameters that need to be referred from literature or determined from complex system identification, which limits the prediction accuracy and efficiency when used for assistive or rehabilitative device control.

In recent years, machine learning methods have been widely applied to achieve promising performance in building the mapping between neuromuscular signals and human joint mechanical functions, including discrete motion pattern recognition based on sEMG signals (Bitzer and Van Der Smagt, 2006; Du et al., 2010; Khokhar et al., 2010; Huang et al., 2011; Duan et al., 2015; Atzori et al., 2016; Hu et al., 2018; Simao et al., 2019; Too et al., 2019; Chen et al., 2020; Coskun et al., 2021) and US signals (Sikdar et al., 2013; Akhlaghi et al., 2016; Huang et al., 2017; McIntosh et al., 2017; Dhawan et al., 2019; Yang et al., 2019; Rabe et al., 2020), as well as continuous kinematics or kinetics prediction (also known as regression problems) based on sEMG signals (Zhang et al., 2012; Zhou et al., 2019; Zhang et al., 2020a; Wang et al., 2021; Yu et al., 2021; Zhang et al., 2022a) and US signals (Guo et al., 2013; Cunningham and Loram, 2020; Zhang et al., 2020a; Jahanandish et al., 2021; Zhang et al., 2022a). Most of the conventional machine learning methods above relied on manually selected low-dimensional explicit features from either sEMG or US signals. For example, the most common time domain and frequency domain features of sEMG signals include mean absolute value, wavelength, root mean square, zero crossing, slope sign change, mean frequency, medial frequency, mean power, and frequency ratio (Phinyomark et al. 2012), while most common architectural and functional features of US signals include pennation angle, fascicle length, muscle thickness, fascicle orientation, tissue displacement, tissue strain, and echogenicity (Hodges et al., 2003; Arampatzis et al., 2006; Shi et al., 2008; Guo et al., 2010; Damiano et al., 2013; Strasser et al., 2013; Hodson-Tole and Lai, 2019; Sheng et al., 2019; Zhang et al., 2020b).

However, the aforementioned low-dimensional explicit features are either too presumptuous or lack meaningful information, which usually causes non-comprehensive sampling of the state-function space on the targeted joint. In addition, as mentioned by Saxby et al. (2020), the processing of US imaging data to derive the aforementioned explicit features is relatively subjective, time-consuming, and tedious. Therefore, more advanced deep learning approaches have been developed to deal with the high-dimensional explicit and/or implicit features from either sEMG or US signals for continuous joint kinematics and/or kinetics estimation (indicating human motion intent or volitional effort) (Cunningham et al., 2017b; Liu et al., 2019; Cunningham and Loram, 2020; Ma et al., 2020; Xu et al., 2020; Yu et al., 2020; Pancholi et al., 2021). For example, in Xu et al. (2020), parallel convolutional neural networks (CNNs) were used to continuously predict an individual finger’s force in real-time based on the temporal energy heatmap and frequency spectrum map from high-density sEMG signals, which outperformed the prediction performance by using a motor unit decomposition method and a conventional sEMG amplitude-based method. Yu et al. (2020) proposed a stacked autoencoder-based deep neural network to continuously estimate wrist kinetics from multiple degrees of freedom with the high-density sEMG signals, where the estimation performance was better than linear regression and support vector regression methods. In Cunningham and Loram (2020), the CNNs-based deep learning approach was implemented to map individual US image frames with a contextual reference frame (prior) to absolute (drift-free) muscle states, including ankle joint angle, joint moment, and sEMG signals from plantarflexor muscles.

The majority of aforementioned contributions by using deep learning methods focused on sEMG-based upper limb kinematics or kinetics estimation problem, and few studies investigated a similar topic for lower limb functional tasks. Although sEMG has been widely implemented to non-invasively measure muscle contraction and thus estimate the volitional effort or motion intent, some inherent challenges still exist, including high noise, cross-talk between adjacent muscles, the inability to measure deep muscle, and sensitivity to the electrode position. In contrast, US imaging provides the direct visualization of the targeted muscle with a high signal-to-noise ratio and can be used to measure muscles in different depths, which would be potentially promising when used for the closed-loop control of rehabilitative or assistive devices. Therefore, the motivation of this work is to investigate the continuous joint volitional effort prediction for lower limb joint functionalities by using high-dimensional features from US imaging and a deep learning method. To the best of our knowledge, only a few recent studies have investigated deep learning approaches for continuous ankle joint kinematics, kinetics, and muscle state estimation (Cunningham et al., 2017b; Cunningham and Loram, 2020). However, they only focused on the active and passive ankle joint movement tasks at the standing posture, and no functional dynamic locomotion tasks were discussed.

In this article, we investigate the feasibility of using high-dimensional US imaging signals and a deep CNN approach to predict the continuous ankle joint net plantarflexion moment during versatile walking tasks. The focus is to verify the robustness of the proposed approach when incorporating new data from various walking scenarios for the personalized prediction of net plantarflexion moment. Unlike features extraction methods in our previous studies (Zhang et al., 2020a,b; Zhang et al., 2021; Zhang et al., 2022a,b), this deep CNN approach does not require the extraction of conventional explicit features for establishing the mapping between skeletal muscle’s US imaging signals and human joint mechanical functions. The implicit features would significantly increase the dimension of the neuromuscular features, which is beneficial for bridging the mapping relationship without losing a large quantity of necessary information.

This article is organized into five sections. Section 2 presents the basic CNN-based deep learning background, the experimental setup for treadmill walking tasks, data collection, deep CNN construction, and statistical analysis. The results of the deep CNN approach-based human ankle joint net plantarflexion moment prediction and its comparison between sEMG and US imaging are presented in Section 3, followed by an interpretation of results, discussion, and potential implementations in Section 4. The conclusion is given in Section 5.

## 2. Methods

### 2.1. Basic background of CNN-based deep learning

In the past decade, CNN has had significant innovations and results in a multitude of fields, such as pattern recognition, classification, and regression problems, from image processing to voice recognition (O’Shea and Nash, 2015). One prominent property that CNN possesses is that it can simplify the input by utilizing various kernel filters for quick processing while maintaining or even amplifying crucial information necessary for recognition. Compared to the traditional artificial neural network (ANN), CNN specializes in learning significant patterns from massive data with fewer parameters. This encouraged researchers and developers to employ CNN to solve complex tasks with larger models that are unable to be properly addressed with classic ANNs.

The design of the training network is a crucial factor in determining CNN’s prediction power. The training network takes input data, interprets it in the hidden layers, and outputs the prediction value through a fully-connected output layer. The CNN output will be compared with the ground truth, and the discrepancy would drive the CNN to adjust the layer parameters to minimize the discrepancy. The filters embedded in CNN can reduce the processing time but still ensure key features of the image are maintained for accurate prediction. The layer types in CNN are usually categorized into convolution, pooling, rectified linear activation (ReLU), batch normalization, dropout, and fully connected (FC) layers. Layer types are briefly discussed below.

The kernel functions, embedded in the convolution layer, filter the pixels in the original image to get various key features/patterns to obtain a tensor-shaped input (Yamashita et al., 2018). In this way, a correlation between the output (i.e., the joint moment) and features of the image is established through CNN. The kernel may move more than one pixel per step when it scans the original image, and the step size is called stride. The selection of the stride value should consider the trade-off between the fineness of the features and the size of the input tensor, which is proportional to the complexity of the designed CNN. A padding operation may be applied within the convolution layer’s parameters to alter the size of the output image by adding rows and columns of zeros on each side of the input (Yamashita et al., 2018). In this way, the data point on the borders can also be taken into consideration. The pooling layer, similar to the convolution layer, extracts significant features but reduces the size and computational power required for data processing (O’Shea and Nash, 2015). The pooling layer can be implemented as an average pooling layer or max pooling layer. Average pooling, the method used in this article, returns the average of all the pixels within the kernel. Max pooling returns the maximum value of all the pixels within the kernel. With more layers, more features are extracted at the expense of extra computational time (O’Shea and Nash, 2015). The ReLU layer replaces negative values with zeros to remove non-linearities and allow for sparsity. Sparsity reduces the time required for training the model and ensures that the neurons are more specialized. Batch normalization reduces the time of the neural network initialization. This process functions by normalizing the mean and variance of the layers, reducing the dependence of gradients, and allowing higher learning rates. A higher learning rate correlates to reduced processing time (Ioffe and Szegedy, 2015). The dropout layer, which is placed after the final ReLU layer, sets input elements to zero at random with a designated probability. The purpose of this layer is to prevent the model from overfitting. The FC is the final layer, which takes the information from the prior layer and turns it into a vector for classification or regression problems.

### 2.2. Subjects for experimental walking study

The study was approved by the Institutional Review Board (IRB) at North Carolina State University (Approval number: 20602). Eight young participants (5 M/3 F) without any neurological disorders or orthopedic impairments, were recruited in this study. The details of anthropometric characteristics for each participant are shown in Table 1. Every participant was familiarized with the experimental procedures and signed an informed consent form before participating in the experiments.

**Table 1.** Anthropometric characteristics (mean and one standard deviation [SD]) of eight young participants

Participant	Sub01	Sub02	Sub03	Sub04	Sub05	Sub06	Sub07	Sub08	Mean	SD
Height (m)	1.93	1.64	1.76	1.77	1.77	1.79	1.72	1.80	1.77	0.08
Mass (kg)	108.0	54.0	84.0	82.0	62.0	70.0	72.0	77.0	76.13	16.29
Age (Years)	24	24	30	27	22	25	26	29	25.87	2.69

### 2.3. Experimental protocol, data acquisition and pre-processing

**Figure 1.**
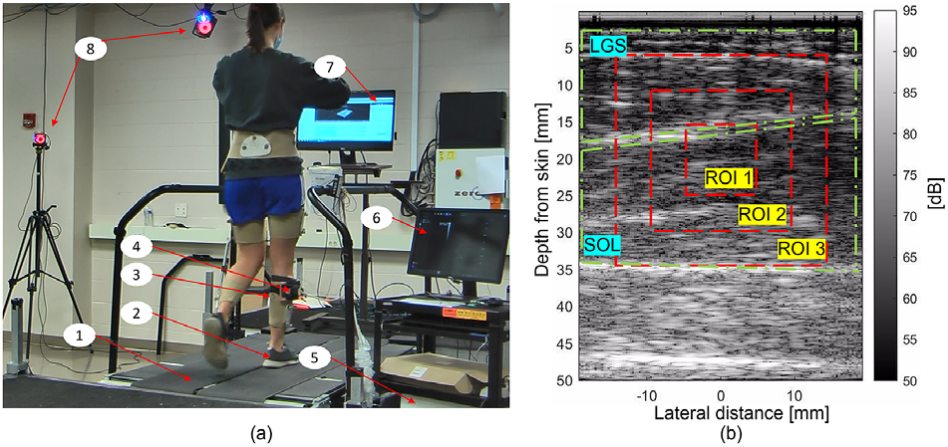
Experimental setup of treadmill walking. (a) Illustration of treadmill walking experimental setup. The walking was performed at speeds of 0.50, 0.75, 1.00, 1.25, and 1.50 m/s. (1) Instrumented treadmill containing two split belts and in-ground force plates. (2) Participants’ lower body with 39 retro-reflective markers attached for kinematics measurements. (3) Three sEMG channels to measure activities of LGS, MGS, and SOL muscles. (4) An ultrasound transducer for imaging of both the LGS and SOL muscles within the same plane. (5) The ultrasound imaging machine for collection of the ultra-fast radio frequency data. (6) A computer screen to show brightness mode (B-mode) US imaging. (7) Computer screen to show live markers and segment links of the participant. (8) 12 motion capture cameras to track markers’ trajectories. (b) A representative B-mode US image with both LGS and SOL muscle in the same plane, as indicated within the upper and lower polygons. The lateral direction is the distance away from the US transducer longitudinal center, and the axial direction is the depth from the skin surface. Three red dashed square areas represent the three regions of interest with a size of 100 × 100, 200 × 200, and 300 × 300 pixels.

Figure 1(a) shows the demonstration of the experimental setup. The treadmill walking data collection process involved the participants walking at speeds of 0.50, 0.75, 1.00, 1.25, and 1.50 m/s. The experiment consisted of an instrumented treadmill (Bertec Corp., Columbus, OH) with two separate belts, and each belt was mounted with a force plate (AMTI, Watertown, MA) to measure the ground reaction force (GRF) signals. Prior to the experiment, 39 retro-reflective markers were placed on the participant’s lower extremities and pelvis to track the movement of each segment. The movements of retro-reflective markers were captured by 12 motion capture cameras (Vicon Motion Systems Ltd, Los Angeles, CA). To measure electrical and architectural muscle activities of plantarflexors, both sEMG sensors (SX230, Biometrics Ltd, Newport, UK) and a US transducer (L7.5SC Prodigy Probe, 6.4 MHz center frequency, S-Sharp, Taiwan) were placed on the surface of plantarflexors of the right leg. We used three channels to measure the sEMG signals from the shank muscles. One channel each was designated for the lateral gastrocnemius (LGS), medial gastrocnemius (MGS), and soleus (SOL) muscles. Close to the placement of sEMG sensors on LGS, the US transducer with a width of 38 mm was attached to the skin through a 3D-printed holder integrated with velcro straps. The transducer was positioned longitudinally and adjusted by observing the brightness mode (B-mode) muscle images on the US machine screen until a clear image that included both LGS and MGS muscles in the same plane was observed. An example of a representative US image in the longitudinal direction that contains both superficial LGS muscle and deep SOL muscle can be seen in Figure 1(b). The US imaging depth was set as 50 mm to include both muscles across all participants.

Under each walking speed, a 3-min walking trial was conducted on each participant, and data within the middle 20 s were collected for processing and analysis. The two channels GRF signals and three channels sEMG signals were sampled and collected at 1,000 Hz by using Nexus 2.9. Also, the 3D coordinates of reflective markers were synchronized and collected at 100 Hz by using Nexus 2.9. For US imaging data acquisition, we applied an ultra-fast frame rate to capture tiny deformation of the targeted muscles’ architecture and facilitate the synchronization with other sensing channels. A trigger signal of 1,000 Hz was sent to the US machine to synchronize the data collection with GRF and sEMG signals, so the plane-wave raw radio frequency (RF) data of the targeted muscles were collected at 1,000 frames per second (FPS).

**Figure 2.**
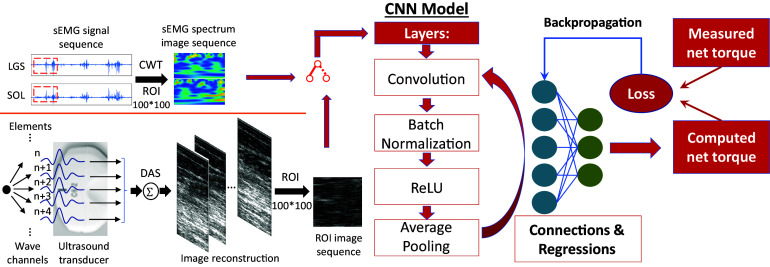
Data collection, pre-processing, and schematic illustration of the proposed deep CNN model calibration. The input to the CNN model includes either the time sequence data of cropped US imaging with different regions of interest or the time sequence data of sEMG spectrum imaging. Thirty-one layers were created in the designed CNN model, including one image input layer at the beginning, one fully connected layer, one regression output layer at the end, and seven sets of intermediate layers. Each intermediate set contained one convolution 2D layer, one batch normalization layer, one rectified linear unit (ReLU) layer, and one average pooling 2D layer.

After data collection, pre-processing procedures were performed before the deep CNN model structure construction, as shown on the left side of Figure 2. To process 3D markers’ coordinates data, the gap-filling was executed in Nexus 2.9, which was followed by inputting both markers’ coordinates and GRF signals to Visual3D (C-Motion, Rockville, MD) for inverse kinematics and dynamics calculation. Raw US RF data were beamformed by using the delay-and-sum (DAS) method (Lu et al., 1994; Thomenius, 1996) to generate the temporal B-mode US image sequence presented in Figures 1(b) and 2. Three regions of interest (ROIs) were selected with a size of 100 × 100 (ROI1), 200 × 200 (ROI2), and 300 × 300 (ROI3) pixels and with the center position at the 0 mm laterally and 20 mm in depth to evaluate the net plantarflexion moment prediction performance by applying the deep CNN model.

In this study, we did not focus on the high-density sEMG singles since only three channels of sEMG signals were collected. First, sEMG signals were run through a band-pass filter between 20–450 Hz. To access the high-dimensional sEMG features, we applied a similar data processing method as mentioned by Chen et al. (2020) to create the sEMG spectrum image sequence for each channel that was synchronized with the US image sequence. To get the comparative ROI as the US image (take ROI1 as the example in this study), data points were segmented in series with a moving window length of 100 points (*n*−99, *n*−98, …, *n*) and a moving step size of 1 point. The data points were then processed through a continuous wavelet transformation (CWT). After CWT, the signals were organized into scale components that contained the frequency-domain information of the time sequence sEMG data. The CWT was calculated with 50 scales obtaining a 50 × 100 matrix at each time point of individual sEMG channels. To have a fair match with the US image, two 50 × 100 matrices from LGS and SOL muscles sEMG channels were merged to get a 100 × 100 matrix at each time instant. The compiled frames from all the data segmentation represent the sEMG spectrum image sequence.

### 2.4. CNN construction and training

This study focused on the stance phase of the gait cycle, where the GRF signals were used to distinguish the heel-strike and toe-off instances during each walking trial with a threshold of 5% body mass. As shown in Figure 2, the input of the CNN is either a US image sequence or an sEMG spectrum image sequence within the stance phase. In the data set used for deep CNN training, a 4:1 ratio was assigned for the training and validation networks. The designed deep CNN model with 31 layers in total was created utilizing Matlab (R2020a, MathWorks, MA). In this study, a customized mini-batch datastore approach was implemented to increase the CNN training efficiency due to the out-of-memory US image data. The mini-batch size was set as 128 for both training and validation procedures on each participant’s data set. At the beginning of each training, the weight parameters of the CNN layers were randomly generated. Then, the parameters were optimized and updated iteratively based on the stochastic gradient descent with a momentum optimizer (Sutskever et al., 2013) and the randomly selected mini-batch size of data points (128) from the training database. Other parameters settings are given as: a maximum number of epochs as 30, initial learning rate as 0.001 and down to 0.0001 after 20 epochs, and validation frequency as training data length/mini-batch. In addition, the training data were shuffled before each training epoch, and the validation data were shuffled before each network validation.

**Figure 3.**
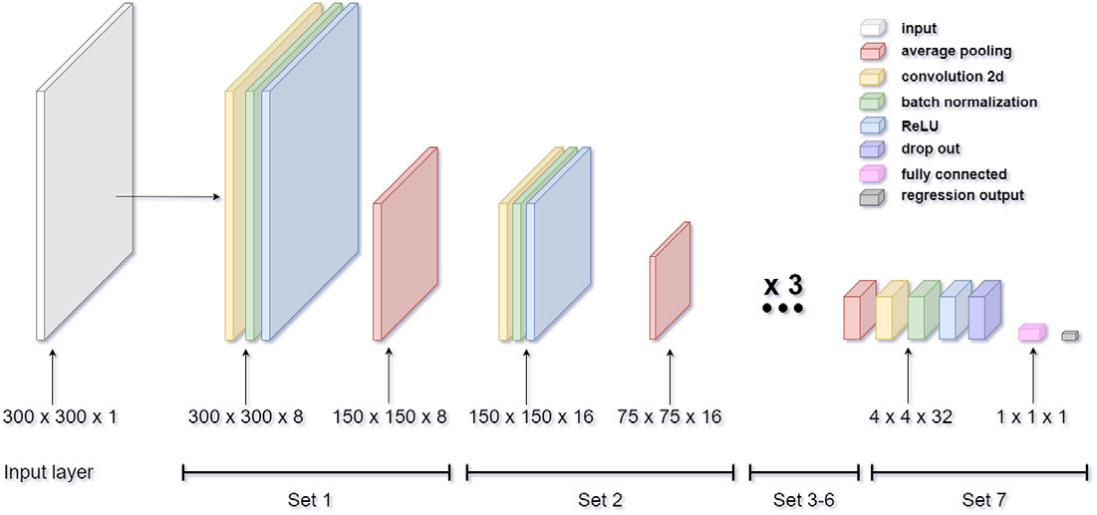
The architecture of the designed deep CNN for the US image and sEMG spectrum image processing. The output size of each layer is based on the input US image’s ROI size of 300 × 300 pixels.

The deep CNN model begins with an image input layer. Then, there are seven sets of layers that contain a convolution, batch normalization, ReLU, and average pooling layer (except for set 7) in respective order, as shown in Figure 3, where an example ROI with the size of 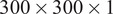 (width × height × depth) was input to the beginning layer. The detailed settings are given below.

The convolution layers across seven sets have different filter numbers of size 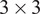. The filter numbers in set 1 and set 2 are selected as 8 and 16, respectively, and the remaining sets have 32 filters. Each convolution layer has a stride of [1 1] and ‘same’ padding. Each average pooling filter has a filter of size 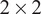, a stride of [2 2], and no padding work throughout the layer sets.

In set 1, the weight and bias of the convolution layer are 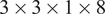 and 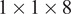, respectively. The batch normalization layer has an offset and scale of 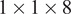. The size of the layers in set 1 remains 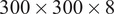 until the average pooling layer where the size shifts to 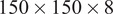.

In set 2, the weight and bias of the convolution layer are 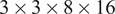 and 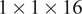, respectively. The batch normalization layer has an offset and scale of 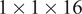. The size of the layers in set 2 remains 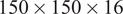 until the average pooling layer where the size shifts to 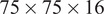.

In set 3, the weight and bias of the convolution layer are 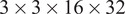 and 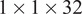, respectively. The batch normalization layer has an offset and scale of 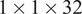. The size of the layers in set 3 remains 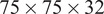 until the average pooling layer where the size shifts to 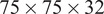.

In set 4, the weight and bias of the convolution layer are 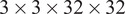 and 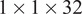, respectively. The batch normalization layer has an offset and scale of 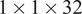. The size of the layer in set 4 remains 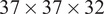 until the average pooling layer where the size shifts to 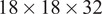.

In sets 5, 6, and 7, the weight and bias of the convolution layer, the offset, and the scale of the batch normalization are all same as in set 4. Finally, the output size of the ReLU layer in set 7 remains 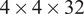. However, instead of an average pooling layer, set 7 contains a dropout layer with a dropout probability of 20% to prevent overfitting, a fully connected layer with the weight and bias of size 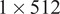 and 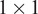, and a regression output layer in respective order. The output of each layer for an exampled US image with an ROI of 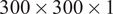 can be found in the Supplementary Material.

**Figure 4.**
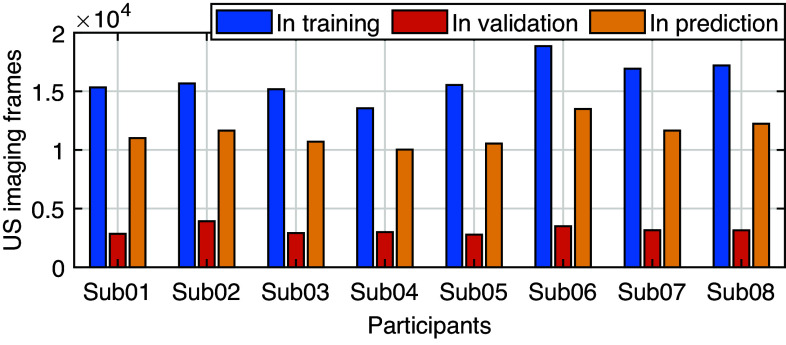
Individual US imaging frames in CNN training, validation, and prediction procedures across five walking speeds.

Under each walking speed, eight gait cycles were randomly selected from the 20 s of collected data. The first five stance cycles were segmented for the deep CNN model training and the last three stance cycles were for prediction analysis. Considering the high difference in plantarflexor muscles’ US imaging among different participants, we focused on the personalized CNN model instead of a generic CNN model across all participants in this study. Therefore, the trained CNN model was not intended to be universal across different participants and walking speeds, but universal across different walking speeds on the same participant. Furthermore, we applied a leave-one-speed-out cross-validation approach to avoid the overfitting issue in the training procedure. Therefore, there were five training models for each participant, and each model contained data in 20 stance cycles (5 out of 8 cycles each speed × 4 speeds) from randomly selected four speeds as the training set, data in five stance cycles (5 out of 8 cycles each speed × 1 speed) from the remaining speed as the validation set, and data in 15 stance cycles (other 3 out of 8 cycles each speed × 5 speeds) as the prediction set. The total US imaging frames that were used in training, validation, and prediction procedures varied for each participant. This is because each participant kept his/her preferred walking cadence on the treadmill at each speed, which is presented in Figure 4. Overall, around 62.5% of the selected data samples were used in the CNN model training and validation while 37.5% were used for the CNN-based prediction of each participant.

### 2.5. CNN performance evaluation and statistical analysis

To evaluate the performance of the CNN approach in terms of modeling the given US imaging data, the regression loss/quadratic loss/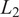 loss, root mean square error (RMSE), and coefficient of determination (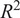) metrics were calculated for both training and prediction procedures, which are formulated as(1)
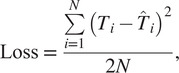
(2)
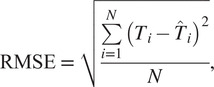
(3)
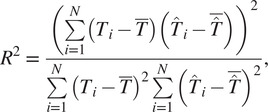
where *N* represents the number of training/prediction data samples, 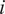 represents the *i*th training/prediction sample in a data set, 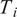 and 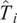 represent the ground truth label and CNN-based calculation for the *i*th training/prediction sample that is obtained from inverse dynamics, respectively. 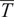 and 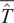 represent the average of the ground truth labels and the average of the CNN-based calculations, respectively. The regression loss is only concerned with the average magnitude of error irrespective of their direction, which has nice mathematical properties that make it easier to calculate gradients. However, due to squaring, calculations that are far away from actual values are penalized heavily in comparison to less deviated calculations. The RMSE serves to aggregate the magnitudes of the errors in calculations for various times into a single measure. RMSE depicts the CNN model’s accuracy and helps in comparing forecasting errors. In the prediction, RMSE values were normalized to individual peak net plantarflexion moment from ground truth labels, noted as N-RMSE.

The normality of the prediction N-RMSE, 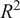, and time cost per image data sets across all participants was tested based on the Shapiro–Wilk parametric hypothesis test (SW test). According to the results from the SW test, a one-way repeated-measure analysis of variance (ANOVA for normal distribution) or Friedman’s tests (for not normal distribution) followed by Tukey’s honestly significant difference tests (Tukey’s HSD) was applied to evaluate the CNN-based prediction performance across all speeds but with different ROIs. Similarly, to evaluate if there was a significant difference between deep CNN-based predictions with US imaging data and sEMG spectrum imaging data with the same ROI size, a paired 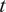-test (normal distribution) or a Wilcoxon signed rank test (not normal distribution) was applied. The significant difference level was chosen as 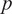 < .05 for all statistical tests.

## 3. Results

### 3.1. Progress during CNN training

**Figure 5.**
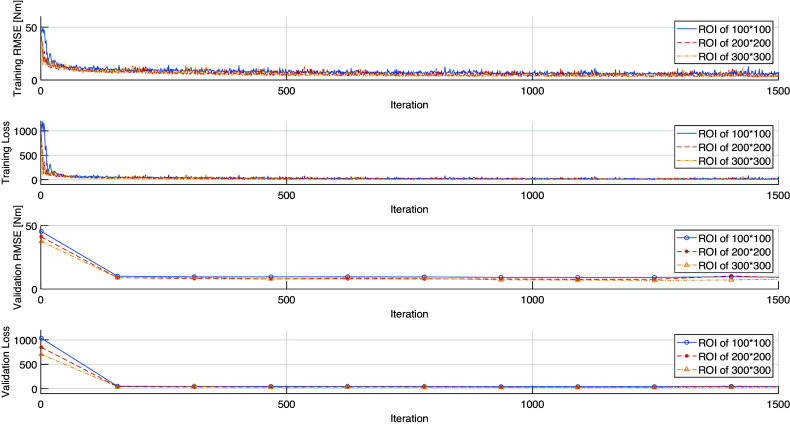
The convergence performance of US imaging-based net plantarflexion moment RMSE and loss with the increase of iteration number during the CNN training and validation procedures. The data set shown here is from Participant Sub08.

With the designed CNN layer number and structure of each layer, the training procedures with different US image’s ROIs converged to a small loss threshold for each participant. By taking Participant Sub08 as an example, the upper two plots and lower two plots in Figure 5 show the loss and RMSE convergence results during the CNN training and validation procedures. It can be observed that after around 300 iterations, the training loss reached a very small value and it did not decrease significantly even though the iteration number increased. It is also observed that a larger ROI resulted in faster convergence speed as well as a smaller steady threshold. Ultimately, the validation RMSE values converged to 13.75, 12.93, and 11.96 Nm, with the ROIs of 100 × 100, 200 × 200, and 300 × 300 pixels, respectively. These results indicate that the larger ROI would be beneficial for better net plantarflexion moment prediction performance. Correspondingly, with the same US imaging frame number, the time cost for the CNN training varies significantly due to the selection of different ROIs. With the aforementioned US imaging frame numbers, as shown in Figure 4, across eight participants, the CNN training times are 3.82 ± 1.30 min, 8.47 ± 2.46 min, and 17.70 ± 3.77 min for ROIs of 100 × 100, 200 × 200, and 300 × 300 pixels, respectively.

### 3.2. Personalized CNN-based net plantarflexion moment prediction performance

**Figure 6.**
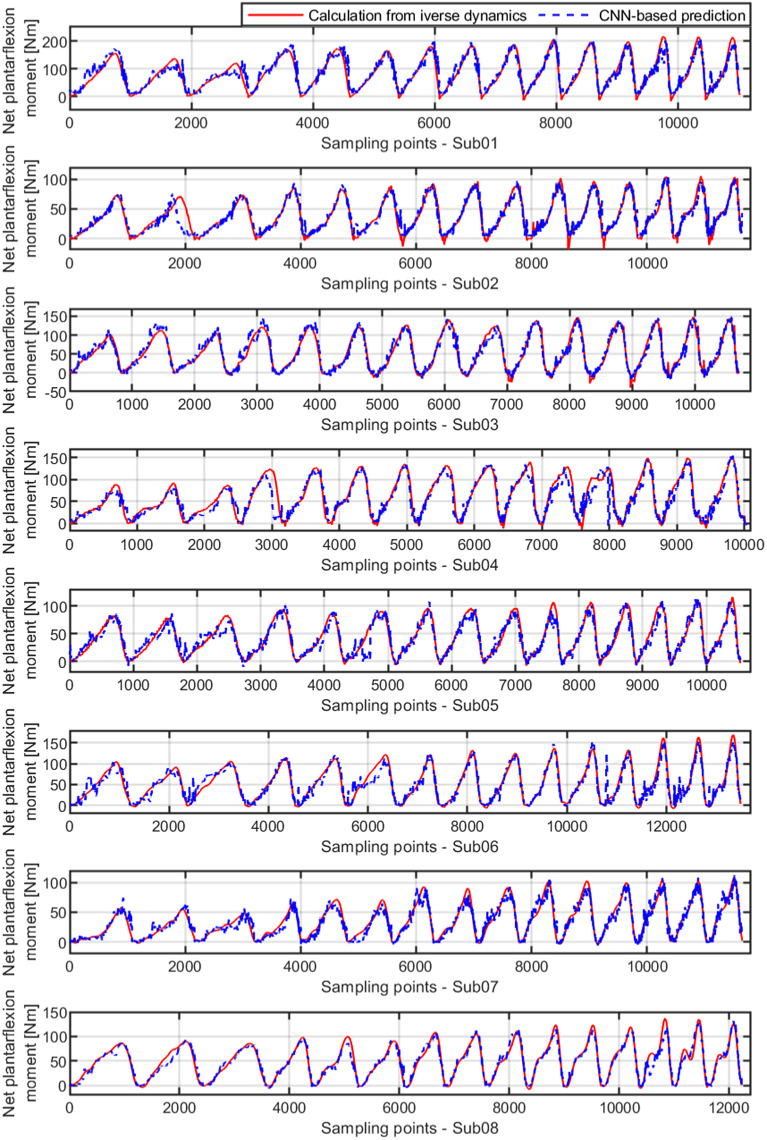
Ankle joint net plantarflexion moment prediction time sequence on each participant by using US images with ROI of 100 × 100 pixels and the deep learning approach. The red solid and blue dashed curves represent the measurements from inverse dynamics and prediction from the CNN model. For each walking speed, three walking stance cycles are included for prediction, therefore, 15 periodic curves are shown for each participant (with the speed order of 0.50, 0.75, 1.00, 1.25, and 1.50 m/s).

**Figure 7.**
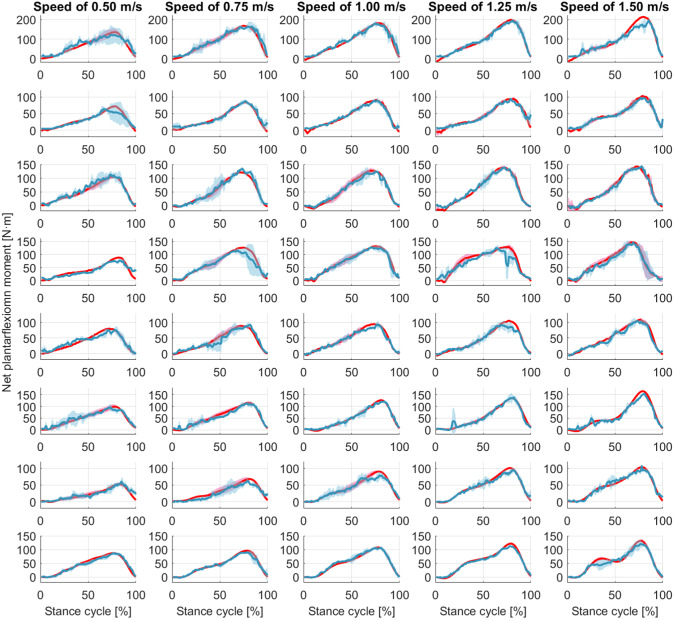
Ankle joint net plantarflexion moment prediction as a percentage of the stance cycle (0% for heel-strike and 100% for toe-off) by using US images with ROI of 100 × 100 pixels and the deep learning approach. The red and blue center curves and shadowed areas represent the mean and standard deviation values (three stance cycles for each curve) of the ground truth and CNN model-based prediction, respectively. Each row subplots represent data from individual participant while each column subplots represent individual walking speed out of five.

**Figure 8.**
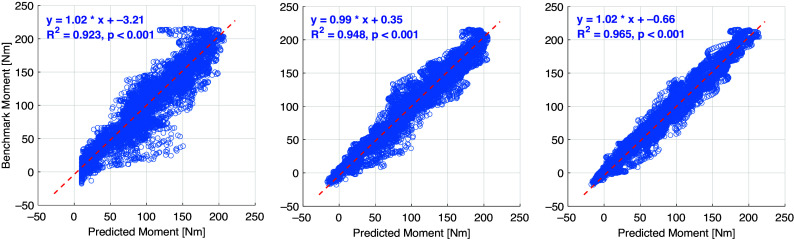
Scatter plots between the net plantarflexion moment benchmark and CNN-based prediction from Sub01 by using ROIs of 100 × 100 (left), 200 × 200 (middle), and 300 × 300 (right) pixels.

Once the personalized CNN model was trained with data sets from all five walking speeds for each participant, new data sets from each walking speed not involved in the training procedure were used to verify the effectiveness and robustness of the personalized CNN model, known as the inter-speed prediction performance. Figure 6 shows the continuous CNN-based prediction with the ROI of 100 × 100 pixels and the ground truth from inverse dynamics individually, represented by blue dashed and red solid curves, respectively. From left to right, every three ramp-up-down curves are from one walking speed out of the five. As the walking speed increased, the peak net plantarflexion moment increased, but the sampling points that were used in the prediction decreased. Therefore, the curves for both ground truth and CNN-based prediction become denser temporally. After separating the prediction performance under each walking speed and normalizing the sampling points throughout each walking stance cycle, the net plantarflexion moment prediction performance corresponding to Figure 6 is demonstrated in Figure 7, where the red and blue center curves and shadowed areas represent the mean and standard deviation values (three stance cycles for each curve) of the ground truth and CNN model-based prediction, respectively. Each row subplots represent data from individual participant while each column subplots represent individual walking speed out of five. Taking results from Sub01 as an example, with the three ROIs, the scatter plots between the ground truth and the CNN-based prediction are shown in Figure 8, where the red dashed line on each plot represents the 45-degree (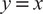) line. It is observed that all data points are distributed along the 45-degree line, indicating a highly linear relationship between the CNN-based prediction and ground truth. A linear regression model was conducted for each ROI, and the coefficients of slope and y-intercept are labeled on each plot. Promisingly, all slope values are very close to 1 (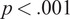) while all y-intercept values are very close to 0 (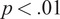). The 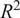 values between the prediction and ground truth are 0.923, 0.948, and 0.965, respectively. Similar results are also observed from data analysis on other participants across multiple walking speeds.

Table 2 summarizes the linear regression results between the CNN-based net plantarflexion moment prediction and the ground truth from each participant with different ROIs. All standard errors of the slope coefficients are less than .005 with the 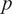-value from the 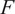-statistic less than 0.001. All standard errors of the *y*-intercept coefficients are less than 0.32 but with a higher variation of 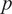-value from the 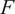-statistic. The goodness of the linear regression is verified with pretty high 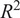 values (0.835–0.965) for all participants. Expect for Sub04 with all three ROIs, Sub05, Sub06, and Sub07 with ROI1, all 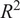 values between the CNN-based prediction and ground truth are higher than 0.9.

**Table 2.** Results of linear regression analysis between net plantarflexion moment CNN-based prediction and ground truth with different ROIs, including mean, standard error (SE), and p-value of slope and *y*-intercept coefficients, as well as R^2^ values

		Slope			*y*-intercept			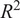
Participants	ROI	Estimate	SE	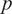 -value	Estimate	SE	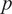 -value	
Sub01	1	1.021	0.003	<0.001	−3.214	0.316	<0.001	0.923
	2	0.994	0.002	<0.001	0.347	0.224	0.240	0.948
	3	1.022	0.002	<0.001	−0.659	0.147	<0.001	0.965
Sub02	1	1.011	0.003	<0.001	0.321	0.132	0.043	0.913
	2	1.021	0.003	<0.001	0.530	0.129	0.051	0.934
	3	1.016	0.003	<0.001	0.158	0.159	0.628	0.947
Sub03	1	0.992	0.002	<0.001	0.409	0.182	0.227	0.926
	2	0.987	0.002	<0.001	1.225	0.120	<0.001	0.949
	3	0.993	0.002	<0.001	1.198	0.139	<0.001	0.957
Sub04	1	1.001	0.004	<0.001	3.524	0.290	<0.001	0.835
	2	0.993	0.003	<0.001	5.627	0.211	<0.001	0.868
	3	0.988	0.003	<0.001	3.223	0.284	<0.001	0.896
Sub05	1	1.021	0.003	<0.001	0.269	0.142	0.111	0.855
	2	1.012	0.003	<0.001	1.153	0.165	<0.001	0.908
	3	1.036	0.003	<0.001	1.182	0.175	<0.001	0.921
Sub06	1	1.009	0.003	<0.001	−0.395	0.132	0.571	0.870
	2	1.012	0.003	<0.001	2.647	0.144	<0.001	0.936
	3	0.998	0.002	<0.001	2.157	0.171	<0.001	0.943
Sub07	1	0.986	0.003	<0.001	1.797	0.155	<0.001	0.883
	2	0.964	0.002	<0.001	2.216	0.069	<0.001	0.911
	3	0.993	0.002	<0.001	2.783	0.076	<0.001	0.931
Sub08	1	1.008	0.002	<0.001	0.469	0.120	0.005	0.928
	2	1.019	0.002	<0.001	0.674	0.112	<0.001	0.936
	3	0.993	0.002	<0.001	0.643	0.176	0.002	0.942

**Figure 9.**
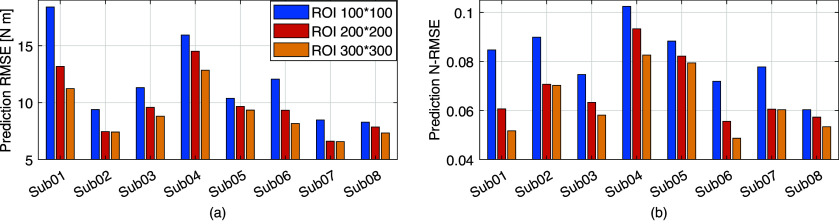
The individual net plantarflexion moment prediction RMSE and N-RMSE values of 15 stance cycles across five walking speeds by using the trained personalized CNN model with different ROIs.

For each ROI, one prediction RMSE value was calculated across 15 stance cycles used in the prediction procedure on each participant to quantitatively assess the accuracy and robustness of the CNN-based net plantarflexion moment prediction by using B-mode US imaging regardless of walking speeds, and the results are presented in Figure 9(a). It is observed that, for the same participant, the increase of the ROI size can moderately decrease the prediction RMSE, although the decrease is minor between ROI 200 × 200 and ROI 300 × 300 on Sub02 and Sub07. However, due to the high variance of subjective peak net plantarflexion moment, using the CNN-based prediction RMSE values to directly evaluate the inter-subject performance is not feasible. Therefore, the relative prediction error, defined as the ratio between the prediction RMSE value and individual peak moment (N-RMSE), was calculated and shown in Figure 9(b). Notably, among all inter-speed predictions, 95.8% (23 out of 24) cases exhibited less than 10% N-RMSE values regardless of the ROI size.

### 3.3. Influence of US imaging ROI size on CNN-based prediction

**Figure 10.**
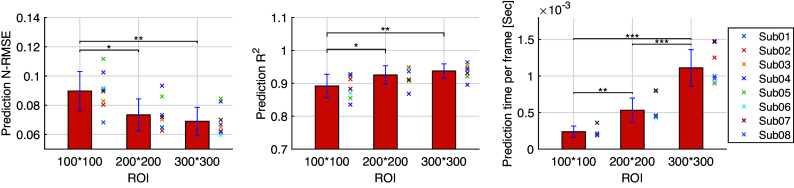
CNN-based net plantarflexion moment prediction results summary across eight participants. Left – Prediction RMSE values normalized to corresponding peak plantarflexion moment, Middle – 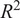 values between net plantarflexion moment prediction and ground truth observation from inverse dynamics, Right – Prediction time cost for each US image frame. Asterisks *, **, and *** represent the statistically significant difference levels at *p <* .05, *p <* .01, and *p <* .001, respectively.

The inter-subject analysis of the US imaging ROI size’s effect on the CNN-based net plantarflexion moment prediction is detailed in this subsection, including the prediction N-RMSE, prediction 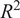, and the time cost per US image during prediction. Figure 10 depicts the inter-subject results of the above evaluation metrics, where each point on the right side of each bar represents a data sample from each participant. Results from the SW test showed that every data group followed a normal distribution with different ROIs. All prediction N-RMSE values are less than 11.20% with these three ROIs, indicating a successful prediction performance by using the deep learning approach. Compared to ROI1, ROI2 and ROI3 significantly reduced the N-RMSE values by 18.17% (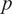 = .024) and 22.97% (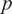 = .004), respectively. However, there was no significant difference between ROI2 and ROI3 (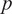 = .727). As mentioned before, all 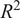 values are higher than 0.9 except for Sub04 with all three ROIs, Sub05, Sub06, and Sub07 with ROI1. Compared to ROI1, ROI2 and ROI3 significantly increased the 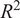 values by 3.78% (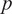 = .042) and 5.14% (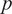 = .009), respectively. However, no significant difference was observed between ROI2 and ROI3 (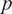 = .687). Except for the prediction accuracy and robustness, the prediction time cost per US imaging frame is essential for implementing the real-time control of robotic rehabilitative or assistive devices. Results in Figure 10 show that the time cost per frame is less than 1.5 ms regardless of the ROI size, which is potentially fast enough for closed-loop control of robotic devices. Furthermore, compared to ROI1, ROI2, and ROI3 significantly increased the prediction time cost per image frame by 223.80% (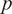 = .009) and 461.97% (*p <* .001), respectively. In addition, ROI3 significantly increased the time cost by 206.42% (*p* < .001) when compared to ROI2. The results showed that the prediction efficiency was more sensitive to the ROI size than the prediction accuracy.

### 3.4. Prediction performance comparison between US imaging and sEMG

**Figure 11.**
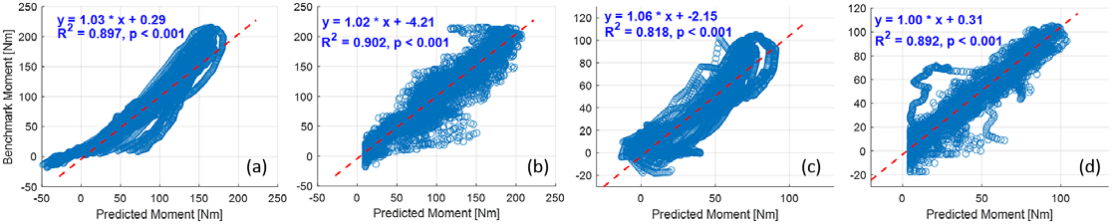
Comparative results of scatter plots between the net plantarflexion moment benchmark and CNN-based prediction: (a) with sEMG spectrum image on Sub01; (b) with US image on Sub01; (c) with sEMG spectrum image on Sub02; (d) with US image on Sub02.

**Figure 12.**
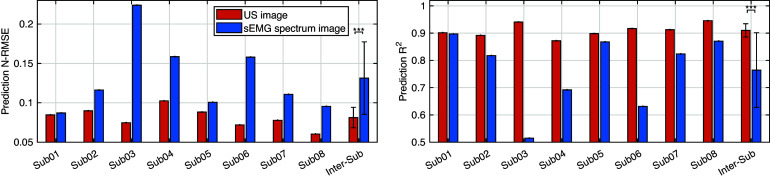
Comparative results of the net plantarflexion moment prediction N-RMSE and 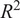 values by using the proposed deep CNN architecture and the same ROI size US image and sEMG spectrum image.

The deep CNN model training and prediction procedures concerning the sEMG spectrum image sequence were similar to the US image sequence with the ROI of 100 × 100 pixels. Similar to the results presented in Figure 8, the comparative scatter plots between the benchmark and predicted net plantarflexion moment values from two representative participants are shown in Figure 11. We found that with the same constructed CNN architecture, the net plantarflexion moment prediction performance is superior to using the US image than the sEMG spectrum image with the same ROI. The higher 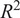 values on these two participants when using the US image indicate a stronger linear relationship between the net moment benchmark and CNN-based net moment prediction. The individual and inter-subject prediction N-RMSE and 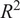 values are presented in Figure 12. Except for Sub01, results from all other participants consistently support the hypothesis that US image + CNN significantly outperforms sEMG + CNN regarding net plantarflexion moment prediction across versatile walking speeds. Across eight participants, statistical analysis shows that with the same ROI and the same CNN architecture, US images significantly reduced the prediction N-RMSE values by 37.55% (*p <* .001) and increased the prediction 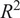 values by 20.13% (*p <* .001) when compared to sEMG spectrum images.

## 4. Discussions

During the past decade, deep learning and CNNs have revolutionized many fields of machine learning, including speech/language recognition, computer vision, biological disease diagnosis, and robotics. Thus, there is no doubt that they may improve the analysis of medical imaging and contribute to incorporating medical imaging into the closed-loop control of biomedical robotic devices. From the perspective of rehabilitation engineering, deep learning and CNNs will be beneficial to bridge the gap between the rehabilitative or assistive device market (that requires intuitive and transparent control methods based on human motion intent or volitional efforts prediction) and recent scientific research results in exoskeletons and prostheses (that show that sEMG-based or US imaging-based proportional control is possible).

### 4.1. Results interpretation

In this article, the investigation tested the hypothesis that the joint net moment is encoded instantaneously with the 3D muscle collagenous structure and is observable using 2D B-mode US imaging. We used the ultra-fast US imaging data collection to generate 26,570–35,838 US images from each of eight participants (in a total of 244,753 images) containing dynamic architectural changes of LGS and SOL muscle during walking at five different speeds. We introduced a framework for the application of CNN-based deep learning to the continuous prediction of ankle joint net moment during treadmill walking at multiple speeds by using those recorded US imaging, and we evaluated the performance of using different sizes of ROI and compared the results with sEMG spectrum imaging with the same size of ROI. When the proposed US imaging + deep learning approach was used, ROI1, ROI2, and ROI3 displayed a prediction RMSE range of 8.48–19.71 Nm (13.04 ± 3.85 Nm), 6.61–15.94 Nm (10.68 ± 3.17 Nm), and 6.57–14.52 (9.98 ± 2.63 Nm), respectively, a prediction N-RMSE range of 0.068–0.112 (0.089 ± 0.013), 0.063–0.093 (0.073 ± 0.011), and 0.059–0.084 (0.069 ± 0.009), respectively, and a prediction 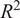 range of 0.835–0.928 (0.892 ± 0.035), 0.868–0.949 (0.925 ± 0.028), and 0.896–0.965 (0.938 ± 0.022), respectively. The results present that the joint net moment of human ankle plantarflexion can be well predicted by the B-mode US imaging signals when people do treadmill walking at different speeds. The joint moment prediction by using the deep CNN model illustrates a good performance across different sizes of US imaging’s ROI, which implies the size of ROI does not play an essential role in the net plantarflexion moment prediction performance, and the deep CNN model is relatively robust among different ROIs, thus we believe that it is a good avenue to explore.

A close look at the net plantarflexion moment prediction performance in Figure 6 presents the subjective smoothness of the deep CNN-based prediction curves. Since an ultra-fast frame rate was employed to collect raw US imaging RF data, tiny muscle deformation was captured across frames, but in the meanwhile, the resolution of each image would be reduced (more noise of each frame) given the center frequency was fixed at 6.4 MHz. One advancement of using deep CNN is that no pre-defined explicit features from US images are required, which usually depends on the high resolution of US images. Therefore, moderate noise in US images due to the high collection frame rate did not impose a noticeable influence on the prediction performance, including timings and peak values. The smoothness issue shown in Figure 6 could be easily addressed by adding a low-pass filter for real-time implementations. One point we would like to make is that the high gait-to-gait and person-to-person variations make it very challenging to build a generalized deep CNN model for all participants. Therefore, in this study, a personalized deep CNN model was trained for each participant and the individual CNN model presented robustness across different walking speeds. The inter-subject analysis in the previous section was based on the individual deep CNN models.

In deep CNN model training, the training accuracy was not sensitive to the increase of the ROI, but the training time was sensitive to the increase of the ROI. When the size of ROI increased from 100 × 100 to 300 × 300 pixels, the average training time increased around 4.6 times (from 3.82 to 17.70 min), although the increase is not linearly related. Impressively, with the deep learning architecture and B-mode US imaging signals, even a fairly small ROI could generate relatively high net moment prediction accuracy (with an average N-RMSE of 8.97%). Regarding the overall accuracy, in this article, the comparison results between US images and sEMG spectrum images with the same size ROI and same deep CNN structure demonstrated the superior net moment prediction performance when B-mode US images were applied. In comparison to existing literature, it is fundamental to note that the results should be compared only with analyses considering the continuous prediction/estimation of joint moment/torque or joint angle by using neuromuscular signals, and the error should be normalized to the corresponding peak value. By using machine learning methods, it is common to see in the literature estimation/prediction error of down to 7–15% (Shi et al., 2008; Dick et al., 2017; Huang et al., 2017; Jahanandish et al., 2019; Zhou et al., 2019; Ma et al., 2020; Jahanandish et al., 2021; Wang et al., 2021; Yu et al., 2021; Zhang et al., 2022a,2022b). However, most of these studies consider using either sEMG or US signals for the upper limb motion or moment estimation/prediction, while a few of them consider the lower limb applications but with relatively simple tasks. Thus, an investigation of complex lower limb tasks, like walking at different speeds, would be reasonable and necessary to justify the proposed B-mode US image + deep CNN approach.

In terms of the ankle joint net plantarflexion moment prediction accuracy, we compared the N-RMSE values between the current study and these established methods in our previous studies (as shown in Table 3), which indicates the superior net plantarflexion moment prediction accuracy by using the proposed US image + deep CNN approach. Compared to the traditional machine learning methods, the deep CNN method achieved many improvements, such as estimation/prediction accuracy, dynamic characteristic, robustness, less pre-processing effort, and more richness of features. In addition, as shown in Figure 10, the time spent per frame during prediction was less than 1.5 ms, which would easily satisfy the real-time application requirement. Compared to the deep CNN-based prediction time, the processing time to obtain B-mode US images online is more significant. It takes around 20–30 ms to generate a B-mode US image based on the build-in online beamforming algorithm, which theoretically indicates the current prediction time is nearly negligible in real-time. As an ongoing project, the real-time implementation of the deep CNN-based net moment prediction is under development.

**Table 3.** The comparison results of the normalized prediction N-RMSE values among different studies

Approaches	HNM (Zhang et al., 2022b)	SVR (Zhang et al., 2022a)	FFNN (Zhang et al., 2022a)
N-RMSE [%]	9.48 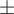 1.51	8.49 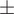 2.74	10.13 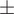 3.44
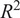	0.91 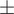 0.04	0.93 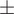 0.05	0.89 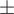 0.08
Approaches	CNN–ROI1	CNN–ROI2	CNN–ROI3
N-RMSE [%]	8.97 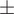 1.33	7.34 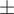 1.09	6.91 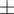 0.95
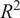	0.89 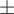 0.03	0.93 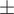 0.03	0.94 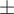 0.02

*Note.* Reported data are the mean ± standard deviation across conditions and participants, where previous studies applied data fusion between sEMG and US imaging signals.

Abbreviation: HNM, hill-type neuromuscular mode; SVR, support vector regression; FFNN, feedforward neural network.

### 4.2. Scientific and clinical significance

The ultimate objective of using the deep CNN-based joint net moment prediction is to design more intuitive and efficient control approaches for wearable assistive robotic devices when considering human volitional contraction efforts. One typical control approach for lower-limb robotic devices to assist human subjects is to measure interaction dynamics or estimate the joint moment from inverse dynamics. However, the nature of interaction dynamics and inverse dynamics may not be appropriate in some cases, as the user must produce a certain torque on the joints to initiate the motion before the devices can generate assistance. If the users are not able to produce sufficient torques on their joints, the robotic devices may not be successfully controlled. Fortunately, this disadvantage could be avoided by using human intent-based control of robotic devices according to biological signals, like sEMG, that are sent from the CNS to the functional motor units. One of the main advantages of using sEMG is the small time difference (between 30–150 ms in Zhou et al. (1995), Lloyd and Besier (2003), and Blackburn et al. (2009)) between signal generation and motion execution, which enables the human intent-based control of wearable robotic devices (the control command generation of the robotic devices advances the generation of the human joint moment or limb motion). Therefore, sEMG signals have been successfully applied in robotic devices control in the past, like examples in Kawamoto and Sankai (2002), Fleischer and Hommel (2008), Lenzi et al. (2012), and Peternel et al. (2016). The crucial perspective of sEMG-based control approaches is that even if the user is not capable of producing sufficient joint motion or joint moment, the motion intent of the human user can still be detected and then the wearable robotic devices can be controlled.

Considering the potential limitations of using sEMG as mentioned in the introduction section, we proposed to use the alternative modality, that is, US imaging, to estimate/predict human volitional/residual joint torque. In our previous study Zhang et al. (2021), we assessed that US imaging-derived signals of the tibialis anterior muscle preceded the ankle joint motion around 45–85 ms. Similarly, the experimental observations from Begovic et al. (2014) showed that the time delay between the onset of muscle fiber motion and the muscle contraction force generation was 49.7 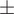 7.0 ms of the quadriceps femoris muscle. Therefore, the time latency between the onset of US imaging-derived signals and the onset of joint torque/motion indicates the comparative joint torque/motion prediction potentials as using sEMG signals. While the CNNs have been applied to many fields as mentioned at the beginning of this section, their application to B-mode US image data from skeletal muscles for a purpose of biomechanics analysis is relatively novel. From the perspective of gait kinematics or kinetics analysis, the standard way to measure the net joint moment depends on the measurement of 3D coordinates of reflective markers via a motion capture system, GRF signals, and inverse dynamics calculations. However, this traditional way encounters several shortcomings: 1) the limitation of instrumental setup in a lab environment and not applicable for field testing, 2) the effort-consuming data collection and post-processing, 3) the challenge of online data acquisition after inverse dynamics, 4) no accessibility of muscle activities during functional tasks. The proposed US image + deep CNN approach provides a forward dynamics approach to estimate/predict net joint moment that has more flexibility in terms of the experimental environment, data collection and analysis, and real-time implementation. Furthermore, the US image + deep CNN approach opens a new gate for sensing volitional user intent and extracting proportional control signals for rehabilitative or assistive robotic devices, including powered ankle-foot prosthesis or ankle exoskeleton, in response to muscle deformations in the shank.

In terms of the control of the powered ankle-foot prosthesis, the net ankle joint moment predicted by using the US image + deep CNN approach could be used to modulate the impedance parameters for the prosthesis, mainly including stiffness and damping coefficients, to imitate the biological ankle joint impedance during the walking stance phase as mentioned in Rouse et al. (2014) and Lee et al. (2016). This has the potential to benefit people with below-knee amputation walking on a treadmill or level ground, such as the increase of gait symmetry, the realization of natural gait patterns, improvement of comfort, and reduction of metabolic cost. In terms of the control of the powered ankle exoskeleton, the net ankle joint moment prediction could be used to adjust the personalized assistance torque profile parameters from the exoskeleton during the walking stance phase, including timings and amplitudes, known as biological mechanism-based control (Nuckols et al., 2021) or proportional sonomyographic control (Dhawan et al., 2019). This manipulation aims to reproduce an individualized assistance torque on the ankle joint that is proportional to the biological torque prediction from the US image + deep CNN approach, as so to enhance the walking function for able-bodied persons, reduce human energy expenditure, or restore normative gait pattern for persons with weakened ankle plantarflexion functions cause by stroke, spinal cord injury, or multiple sclerosis.

Since we focus on the most common and relative complex locomotion tasks, that is, treadmill walking with multiple speeds, and the ankle joint plantarflexion function is essential to provide “push-off” effort during walking, both LGS and SOL muscles were investigated to understand the mapping between the B-mode US images and net ankle joint moment. However, the significance of the current investigation lies in the potential application to estimate/predict other joints’ mechanical functions, as well as to estimate/predict states generally from individual muscles, given the fact that the information is well encoded in skeletal muscles’ collagen structure and are observable by using US imaging (Cunningham and Loram, 2020). Recent research studies have also applied US imaging + deep (machine) learning to estimate skeletal muscles’ activation levels (Cunningham et al., 2017b; Cunningham and Loram, 2020; Feigin et al., 2020), fascicle length (Rosa et al., 2021), fascicle orientation (Cunningham et al., 2017a), and muscle segmentation (Carneiro and Nascimento, 2013; Zhou et al., 2020).

### 4.3. Limitation and future work

Although the proposed US image + deep CNN approach showed promising performance in terms of ankle joint net moment prediction accuracy and time efficiency, some limitations still exist in the current study. First of all, raw US imaging RF data beamforming to B-mode US images, net architecture, and hyperparameter settings seem to be fundamental for the analysis of US image data with deep CNNs, since they can strongly change the final prediction accuracy in the testing image set, and time to converge. However, the aforementioned aspects were pre-selected and no comparison studies between different beamforming algorithms, different net architectures, or different hyperparameter settings were conducted since the focus of the current study was to investigate the feasibility and effectiveness of the US image + deep CNN approach instead of a systematic analysis of various deep CNN models. Secondly, the ultra-fast frame rate was applied to collect the raw US imaging RF data, but B-mode US images at 1,000 FPS were generated offline by using the DAS beamforming method for both deep CNN model training and prediction. However, this ultra-fast frame rate is likely to be down-sampled to 1/30–1/20 for real-time implementation according to the previous discussion. Thirdly, only the plantarflexor muscles were involved in the net plantarflexion moment prediction without considering the effects of antagonistic muscle groups. Lastly, only two sEMG channels were used to collect signals from LGS and SOL muscles and generate spectrum images for comparison with US images. It is hard to specify if the inferior prediction performance of using sEMG spectrum images came from the small number of channels and the noisy nature of sEMG signals.

Our future work will focus on 1) the effects of various hyperparameters in the deep CNN model on the US images-based ankle joint net moment prediction performance; 2) the real-time implementation of the proposed US image + deep CNN approach for ankle joint net moment prediction during treadmill walking at multiple speeds on both neurologically-unimpaired persons and incomplete spinal cord injury/stroke patients; 3) the incorporation of the real-time ankle joint net moment with closed-loop assistance-as-needed control for a cable-driven ankle exoskeleton as mentioned in Zhang (2021) on both subject groups.

## 5. Conclusion

This article proposed the first comprehensive analysis of a novel computational method for estimating human walking volitional effort on the ankle joint using US images of skeleton muscles. Specifically, we investigated the effectiveness and robustness of the B-mode US imaging + deep CNN approach for the ankle joint net moment prediction during treadmill walking at multiple speeds. We evaluated the effects of the US image’s ROI size on the net moment prediction performance and compared the deep CNN-based prediction performance by using B-mode US image data and sEMG spectrum image data with the same ROI size. Results from eight participants without any neurological disorders tested the hypothesis that the joint net moment was encoded instantaneously with the 3D muscle collagenous structure and was observable using 2D B-mode US images. The proposed framework achieved a relatively high net moment prediction accuracy (with an average N-RMSE of 8.97%) even by using a small ROI of 100 × 100 pixels. The prediction time per image was less than 1.5 ms, which could easily satisfy the real-time implementations when incorporated with the closed-loop control of robotic devices. With the same deep CNN structure, the comparison results showed that US images significantly reduced the prediction N-RMSE values by 37.55% (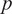 < .001) and increased the prediction 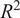 values by 20.13% (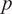 < .001), when compared to sEMG spectrum images. Impressively, the proposed US image + deep CNN approach produced accurate results comparable or superior to the average classical machine learning methods, which suggests that further studies may lead to the improvement of the overall field of US imaging-driven control of the ankle-foot prosthesis or ankle exoskeleton.

## Data Availability

Data availability is not applicable to this article as no new data were created or analyzed in this study.

## Supplementary material

For supplementary material accompanying this paper visit https://doi.org/10.1017/wtc.2022.18. click here to view supplementary material
