# Supplementary material for: A deep learning method to predict ankle joint moment during walking at different speeds with ultrasound imaging: A framework for assistive devices control
Source: Wearable Technol. 2022 Sep 6;3:e20. doi: 10.1017/wtc.2022.18 (PMC10936300; doi:10.1017/wtc.2022.18)
Supplement: Supplementary file 1 [file S2631717622000184sup001.pdf]

Supplemental table of contents

1. Visualization of convolutional neural network layers for one participant at ROI 301 * 301
2. Convolutional neural network structure analysis in Matlab
3. Convolutional neural network Matlab code
4. Videos of brightness mode (B-mode) ultrasound images from two participants during the recorded walking trials at 5 different speeds

1. Visualization of convolutional neural network layers for one participant at ROI 301*301

The following supplemental figure is a visual of the activations of the convolutional neural network (CNN) for layers 2-29. The file contains one representative subject, Sub01 and single region of interest, 301*301. The activation of the CNN can be thought of as the output where each image corresponds to an activation of that layer. The input layer, regression output layer, and fully connected layer are unable to be pictured. The input layer will not contain activations as it is the initial image. The regression output and fully connected layers are vectors that are unable to be visualized. The prefix refers to the layer number, while the suffix refers to the layer type and set number. (example: first relu layer of first set = Layer1_relu_1) The file names can be related to the network architecture in supplemental file X2 and Fig. 3 within the article.

2. Convolutional neural network structure analysis in Matlab

The analyzeNetwork output in Matlab. This figure contains the layer parameters, names, output, and architecture. The network architecture is for Sub01 with a region of interest of 101*101. Visualization of each layer activation or output can be seen in supplemental file X1 and an additional layout of the CNN architecture in Fig. 3 within the article.

3. Convolutional neural network Matlab code

This file contains the PDF version in addition to the code used to create the convolutional neural network. The file pathways on lines 30 and 31 must be adjusted depending on the user. Lines 6-16 must be adjusted depending on the subject, speed, region of interest, and desired filenames to be saved at the end of the code. The files required to run the code are contained in the folders named 'Sub01Files' and 'Sub02Files' for .mat files, and 'SupplementalFileX4' for ultrasound image frames.

4. Videos of brightness mode (B-mode) ultrasound images from two participants during the recorded walking trials at 5 different speeds

This folder contains the videos of ultrasound b-mode images from Sub01 and Sub02 for all walking trials. The images were collected via an ultrasound probe on the gastrocnemius and soleus muscle for 20 seconds during gait at various walking speeds. However, only the videos of each walking trial are provided due to the large memory requirement of all the image frames (around 20000 frames * 10 trials for these two representative subjects). The ultrasound image frames are available upon request from the corresponding author.

All these supplemental materials can be found in the DOI: 10.21227/7beh-f093
